# Antimicrobial Activity of Hybrids Terpolymers Based on Magnetite Hydrogel Nanocomposites

**DOI:** 10.3390/ma12213604

**Published:** 2019-11-03

**Authors:** Reem K. Farag, Ahmed Labena, Sahar H. Fakhry, Gehan Safwat, Ayman Diab, Ayman M. Atta

**Affiliations:** 1 Petroleum Application Department, Egyptian Petroleum Research Institute (EPRI), Nasr City, Cairo 11727, Egypt; reem_kamal.kamel2009@yahoo.com (R.K.F.); labena.labena@googlemail.com (A.L.); 2 Faculty of Biotechnology, October University for Modern Science and Arts, 26 July Mehwar Road intersection with Wahat Road, 6th October City P.O. Box 2511, Egypt; sahar.hossam@msa.edu.eg (S.H.F.); gehan.safwat@hotmail.co.uk (G.S.); aymanalidiab@gmail.com (A.D.); 3 Chemistry Department, College of Science, King Saud University, P.O. Box 2455, Riyadh 11451, Saudi Arabia

**Keywords:** nanocomposite hydrogels, iron oxide nanocomposite, antibacterial, antifungal, swelling

## Abstract

In the past few years, the development of hydrogel properties has led to the emergence of nanocomposite hydrogels that have unique properties that allow them to be used in various different fields and applications such as drug delivery, adsorption soil containing, tissue engineering, wound dressing, and especially antimicrobial applications. Thus, this study was conducted in order to fabricate a novel crosslinked terpolymer nanocomposite hydrogel using the free radical copolymerization method based on the usage of 2-acrylamido-2-methylpropane sulfonic acid (AMPS), acrylamide (AAm), acrylonitrile (AN), and acrylic acid (AA) monomers and iron oxide (Fe_3_O_4_) magnetic nanoparticles and using benzoyl peroxide as an initiator and ethylene glycol dimethacrylate (EGDMA) as a crosslinker. The structure of the synthesized composite was confirmed using Fourier transform infrared (FTIR) spectroscopy and x-ray powder diffraction (XRD) measurements. Furthermore, the surface morphology and the magnetic nanoparticle distributions were determined by scanning electron microscopy (SEM) measurement. In addition, the swelling capacity of the hydrogel nanocomposite was measured using the swelling test. Lastly, the efficiency of the produced composite was evaluated as an antimicrobial agent for Gram-positive and Gram-negative bacterial strains and a fungal strain.

## 1. Introduction

Hydrogels are three-dimensional structure materials that contain hydrophilic polymer chains. Hydrogels are considered useful in several fields due to their tenable chemical, biological, and physical properties. Despite their properties, they have many limitations such as low strain, low thermal stability, and poor mechanical strength that limit their usage in many applications. Due to those limitations, nanocomposite hydrogels have been designed with enhanced and exclusive characteristics [1,2,3]. Nanocomposite hydrogels, called hybrid hydrogels, are known to confer polymeric networks with highly hydrated properties. These nanocomposite hydrogels also possess superior electrical, physical, biological, and chemical properties. Furthermore, they displayed more elasticity than other hydrogels [4]. Due to their enhanced response, action capability at a distance, high strength, and deformability, nanocomposite hydrogels have attracted huge attention related to studying their structural aspects [5]. In order to manufacture nanocomposite hydrogels with improved swelling/deswelling, physical, chemical, and biological properties, organic and inorganic materials are combined [6]. Moreover, nanocomposite hydrogels are known to exhibit various properties such as high heat resistance, high strength, and good modulus [7]. Nanocomposite hydrogels are obtained by the combination of various types of nanoparticles with the polymeric networks. Some examples of that are the carbon-based nanomaterials, polymeric nanoparticles dendrimer (hyperbranched polyesters), metal, or metal oxide nanoparticles (silver, gold, and zinc oxide or iron oxide), and many other examples [8,9]. The polymer nanotechnology in the past has established many nanocomposite hydrogels that are biocompatible and biodegradable to be used in biomedical fields such as in tissue engineering, drug delivery, and antimicrobial applications [10]. The inorganic-based nanocomposite hydrogels exhibited promising results for the inhibition of bacteria such as silver (Ag) nanocomposite hydrogels along with curcumin or β-chitin due to their superior antibacterial properties [11,12,13]. Ag nanoparticles are considered a very useful material against the microorganisms that can be directed to biomedical applications, in addition to it being eco-friendly and non-toxic. Accordingly, the nanocomposite hydrogel was used in wound dressing (wound healing) and antibacterial applications when placed on an infected burn [14].

Iron oxide nanoparticles such as magnetite (Fe_3_O_4_) and maghemite (Fe_2_O_3_) are biocompatible nanomaterials that have been actively investigated for magnetic resonance imaging, stem cell storing and manipulation, drug delivery guiding, and targeted cancer treatment [15,16,17,18]. The iron oxide nanoparticles were inserted inside chitosan/hydroxyapatite to improve their radio-capacity and osteoblast proliferation activities to promote bone healing [19]. The iron oxides also reported as antimicrobial nanomaterials that act via physical damage of the cell membranes [20,21,22]. The fabrication of a chitosan–iron oxide-coated graphene oxide nanocomposite hydrogel as a robust biofilm with antimicrobial activity properties was reported [23]. There has not been any report on the antimicrobial characteristics of the iron oxides polymeric nanocomposite; thus, this research was directed for this purpose in order to test the efficiency of the produced composite as an antimicrobial agent for Gram-positive bacteria, *Bacillus subtilis*, Gram-negative bacteria, *E. coli,* and fungal strain, *Candia albicans.* The dispersion of the iron oxides nanoparticles in the hydrogel composites and in the aqueous solution without agglomerations are big challenges that are solved by selecting the suitable monomers that interacted well with iron oxide during their formation in the nanosizes using an in situ technique [24]. In this respect, the present work aims to use 2-acrylamido-2-methylpropane sulfonic acid (AMPS), acrylamide (AAm), acrylonitrile (AN), and acrylic acid (AA) monomers that can easily interact with the iron oxide nanoparticles and increase their dispersion in the produced hybrid gel composites.

## 2. Experimental

### 2.1. Materials

Acrylonitrile (AN), 2-acrylamido-2-methylpropane sulfonic acid (AMPS), magnetic nanoparticles (Fe_3_O_4_), fluconazole, tetracycline, and amoxicillin antibiotics were obtained from Sigma-Aldrich. Acrylic acid (AA), acrylamide (AAm), nutrient agar, and ethanol (70%) were purchased from Thermo fisher scientific. Benzoyl peroxide (BP) initiator and ethylene glycol dimethacrylate crosslinker (EGDMA) were collected Chembid. Sabouraud Dextrose agar or broth (SDA/SDB) and Mueller Hinton broth or agar (MHB/MHA) were obtained from Difco, Franklin Lakes, NJ, USA.

### 2.2. Techniques

#### Synthesis of Nanocomposite Hydrogel

Eight Fe_3_O_4_–hydrogel nanocomposites were synthesized, and their components were shown in (Table 1). The crosslinked terpolymers either in the presence or absence of iron oxide nanoparticles were prepared using the free radical solution polymerization technique. In this respect, the Fe_3_O_4_–hydrogel nanocomposites were successfully prepared as described in the previous works [25,26]. The monomer weights of AMPS, AA, and AN monomers (summarized in Table 1) were dispersed in water under mechanical stirring and N_2_ atmosphere. The iron oxide added to the reaction mixture with the desired amount of EDGMA crosslinker in the aqueous dispersion with the concentration of 10 wt % from the total weights of the reacted monomers under stirring at room temperature. The reaction temperature increased up to 40 °C and the BP initiator (6 wt % of the total weight of monomers and crosslinker) was added to the reaction mixture. The mixed reaction solutions were refluxed for 3 h under at 85 °C under N_2_ atmosphere. The solid Fe_3_O_4_–hydrogel composites were separated from the reaction mixture by using an external magnet via washing with ethanol two times and furthermore dried in a vacuum oven at 40 °C for 8 h. The first three samples contained the same materials but with different concentrations, and they were named N1, N2, and N3; the fourth sample was the control and named C1. The other three samples contained the same reagents with different concentrations, and they were named X1, X2, and X3 magnetite/terpolymer hybrids, the fourth sample was the control, which was named C2.

### 2.3. Characterization

The infrared spectra of the samples were obtained with a “Perkin-Elmer Spectrum One Fourier transform infrared (FTIR)” Spectrophotometer in the range of 400–4000 cm^−1^. The samples were prepared in a pellet form for the analysis, the samples were diluted with potassium bromide powder (KBr, IR grade Merck) (samples/KBr: 1/200 (w/w). The X-ray diffraction (XRD) patterns of the samples were obtained by using a “Philips analytical X’Pert Pro X-ray Diffract-meter” with CuK α radiation (45 kV, 40 mA, and λ = 1.5406). The surface morphology of the nanoparticles was determined in the samples using the SEM (Philips, Model XL30, 17 kV).

#### 2.3.1. Swelling Behavior

To study the swelling behavior of the nanocomposite hydrogels, the samples were immersed in distilled water for 110 min, and the equilibrium swelling ratio was calculated based on the following equation:

$$\mathrm{Swelling}\;(\%)=\frac{Ws-Wd}{wd}\times 100$$

where *Ws* represents the weight of the sample in swollen condition, and *Wd* represents the weight of the sample in dried condition [27,28].

#### 2.3.2. Biological Activity

The biological activity of the resynthesized samples X1, X2, and X3 magnetite/terpolymer hybrids were tested using the modified agar well diffusion method [19]. Briefly, the tested microbial strains *Bacillus subtilis* (ATCC 6633), *Escherichia coli* (ATCC 8739), and *Candia albicans* (ATCC 10231) were streaked on the nutrient agar (NA) surface, for the bacterial strains and on Sabouraud dextrose agar (SDA) for the fungal strain. Then, using a 10-mm cork borer, two wells were made on the agar plates and 100 µl of the samples (X1, X2, and X3 magnetite/terpolymer hybrids) were added into the wells in addition to the positive control antibiotics, which are fluconazole (100 ppm), tetracycline (100 ppm) and amoxicillin (100 ppm), and the negative control, which is sterile water. Then, the agar plates were left for incubation for 48 h at 30 °C for the fungal strain and for overnight at 37 °C for the bacterial strains. The inhibition zone diameter (mm) was measured in order to determine the antimicrobial activity. The experiment was repeated twice, and the measurement was taken in three different fixed directions [29,30].

#### 2.3.3. Minimum Inhibitory Concentration (MIC) and Minimum Bactericidal/Fungicidal Concentration (MBC/MFC) Measurement

According to Wiegand et al. [31], the minimum inhibitory concentration (MIC) is defined as “the minimum concentration of an antimicrobial agent that inhibits the development of a microbial growth”. Meanwhile, the minimum bactericidal/fungicidal concentration (MBC/MFC) is defined as “the minimum concentration of an antimicrobial agent required to kill 99% of the microbes” [32]. The minimum inhibitory (MIC) and the minimum bactericidal/fungicidal (MBC/MFC) concentrations were determined by using microdilution method in 96-well microtiter plates according to Amesterdam [33]. In this experiment, the bacterial and the *Candida* strains were grown on a Mueller Hinton broth or agar (MHB/MHA) for bacterial strains and Sabouraud dextrose broth or agar (SDB/SDA) for fungal strains. An inoculum suspension was prepared according to Cockerill [34], standard 0.5, and the bacterial inoculate contained 1-2 × 1 magnetite/terpolymer hybrid ^8^ Colony-forming unit (CFU)/mL for Gram-positive bacteria and 1-2X1 magnetite/terpolymer hybride0^9^ CFU/mL for Gram-negative bacteria. Meanwhile, the Candia strain was corresponding to 5X1 magnetite/terpolymer hybride0^6^ CFU/mL; all the method details were previously reported. The nanocomposite samples (X1, X2, and X3 magnetite/terpolymer hybrids) that have shown the highest antimicrobial activity were used to measure the MIC values using the Nunclon^TM 2^ microtiter plates and the two-fold microdilution method. A 200-µL final volume was achieved in the 96-well microtiter plates by first distributing 100 µL of the nanocomposite hydrogel samples in the plates and then inoculating them with 100 µL of the microbial suspension. One column was used as a positive control for all the tested samples where it contained only the media and the inoculum, and another column was used as a negative control where it contained only the media. Lastly, the microtiter plates were incubated aerobically at 37 °C for 20–22 h for bacterial species and at 30 °C for 48 h for the *C. albicans* [30]. The MBC/MFC values of the nanocomposite hydrogel samples (X1, X2, and X3 magnetite/terpolymer hybrids) were detected by taking samples from each well that displayed no visible growth and further subculturing them onto the MHA media and SDA media [35]. Then, the agar plates were incubated at 37 °C for 20–22 h for the bacterial species and at 30 °C for 48 h in the case of *C. albicans* until the microbial growth was detected in the control plates. The MBC/MFC values were defined as “the corresponding concentrations of the nanocomposite hydrogel samples required for killing 99% of the microorganisms” [30].

## 3. Results and Discussion

**Scheme 1:**
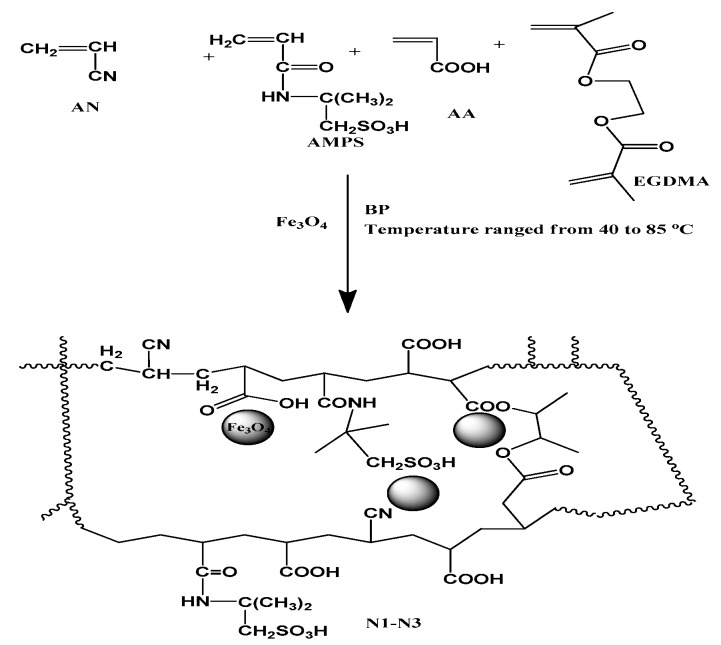
Chemical structure of the N1–N3 magnetite/terpolymer hydrogel nanocomposites.

The aim of this work was to manufacture eight samples of super-absorbed hydrogel nanocomposite based on AMPs, acrylic acid, acrylonitrile monomers, acrylamide monomers, and iron oxide magnetic nanoparticles through using benzoyl peroxide as an initiator and ethylene glycol dimethacrylate (EGDMA) as a crosslinker. The chemical structure of the prepared Fe_3_O_4_/AmPS/AA/AN nanocomposite is selected and represented in Scheme 1. In order to determine the chemical structure of the manufactured nanocomposite hydrogel, linear terpolymers were prepared under the same reaction conditions as the hydrogel but without the usage of the EGDMA crosslinker and the AMPS monomers. 

### 3.1. Characterization of Polymer Composites

**Figure 1:**
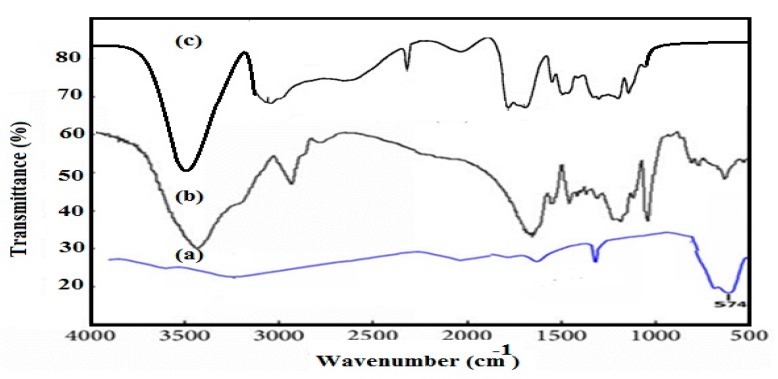
Demonstrates the FTIR spectra where (**a**) represents the iron oxide nanoparticle; (**b**) represents the X1 magnetite/terpolymer hybrid; and (**c**) represents the C1 terpolymer.

The functional groups of the synthesized samples were first determined using the FTIR analysis technique, where it showed that all the samples have the same characteristic bands. Figure 1a–c shows the FTIR spectra of the iron oxide magnetic nanoparticles, X1 magnetite/terpolymer hybrid, and C1 terpolymer that contained one mole of each monomer (acrylonitrile, acrylamide, and acrylic acid) and 10% of the total weight of the iron oxide nanoparticles respectively, which was selected as a representative sample. Figure 1 shows the characteristic bands at 2926 and 2855 cm^−1^, which were assigned to the stretching vibration of the aliphatic C–H bond. On the other hand, the peaks at 1750 cm^−1^ were assigned to the stretching vibration of the C = O, and the peaks at 1143 cm^−1^ were assigned to the stretching of the C–O bond. Furthermore, the 3435 to 3600 cm^−1^ bands were assigned for the stretching of –NH and COOH, respectively. In addition, the disappearance of the (C = C) band at 1650 cm^−1^ confirms the completion of the terpolymerization reaction [36]. The peak at 2441 cm^−1^ in the spectrum of C1 (Figure 1c) could be attributed to the –CN of AN in the terpolymer. Additionally, the additional peak that appeared at 590 cm^−1^ in the spectra of magnetite (Figure 1a) or the X1 magnetite/terpolymer hybrid (Figure 1b) and disappeared in C1 spectra (Figure 1c) corresponds to the formation of Fe_3_O_4_ magnetic nanoparticles as seen, which confirmed the preparation of magnetic nanoparticles hydrogel matrices. These FTIR results significantly agree with Kim et al. [37], where the research was done on polymeric nanocomposite hydrogels and showed that the characteristics peaks appeared at 3390, 3193, 1620 cm^−1^, which is due to the CONH_2_ group as well as the appearance of other characteristics peaks at 1130 and 1062 cm^−1^, which is due to the C–O bond.

**Figure 2:**
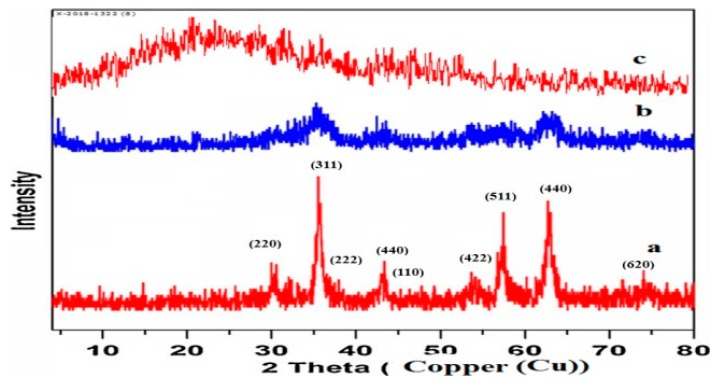
Demonstrates the X-ray diffraction (XRD) results where (**a**) represents the Fe_3_O_4_ nanoparticle, (**b**) represents the X1 magnetite/terpolymer hybrid, and (**c**) represents the control C2 terpolymer, respectively.

The XRD was used as another method for the characterization of the synthesized nanoabsorbents and their magnetic counterparts. The data obtained from the results (Figure 2) of the XRD analysis regarding the nanoparticles, the X1 magnetite/terpolymer hybrid, and the C2 hydrogel terpolymer without the nanoparticles indicated that the X1 magnetite/terpolymer hybrid and C2 hydrogels had a high amorphous structure compared to the linear structure of the iron oxide nanoparticles. The C2 hydrogel and the X1 magnetite/terpolymer hybrid were used as representative samples, respectively, and they are also shown for comparison. The diffraction peaks of the C2 hydrogel are located at 2θ (approximately 25–40°) were very weak, signifying an amorphous structure. The XRD pattern of magnetite (Figure 2a) elucidates the magnetite crystalline structure, and their diffraction peaks match well with the standard magnetite diffraction peaks (JCPDS file No. 19-0629). Whereas the diffraction peaks of the X1 magnetite/terpolymer hybrid are located at 2θ (approximately 15° and 40°) and were strong and intense, signifying that there was an incorporation of a high crystalline structure of iron oxide nanoparticles.

**Figure 3:**
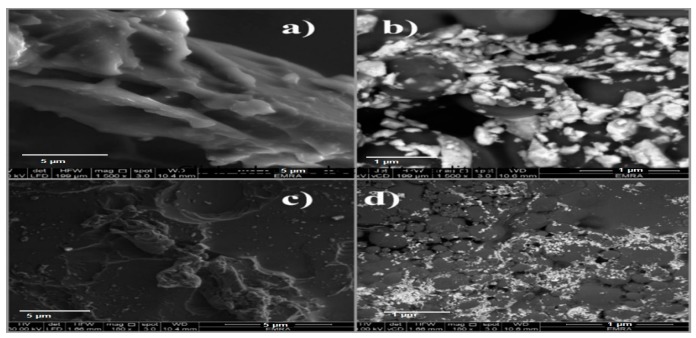
Demonstrates the scan electron microscope images where (**a**) represents the C2 terpolymer, (**b**) represents the X1 magnetite/terpolymer hybrid; (**c**) represents the C1, and (**d**) N1 represents the magnetite/terpolymer hybrid, respectively.

The surface morphology and the distribution of the iron oxide nanoparticles were determined using the SEM equipment. For the SEM analysis, the C1, X1 magnetite/terpolymer hybrid, C2, and N1 were chosen as the representative samples and represented in Figure 3a–d. The C2 and C1 terpolymer micrographs confirm the absence of magnetite that appeared as spherical white nanoparticles in the X1 magnetite/terpolymer hybrid and N1 micrographs nanocomposite terpolymer (Figure 3b,d). The particle sizes of the used magnetite ranged from 15 to 20 nm (as received commercially), and they were all distributed over all the surface of the sample compared to the control, which was the hydrogel without the nanoparticles. The particles were all generally spherical in the shape. Although it can be supposed that the size of the distribution for all the samples were rather wide, it can be seen that the hydrogels have a porous network structure. In the case of the X1 and the N1 magnetite/terpolymer hybrids, the magnetite was found all over the hydrogel surfaces. Moreover, the magnetite nanoparticles were agglomerated on the surface of X1 (Figure 3b) more than N1 (Figure 3d). This observation referred to the presence of the negatively-charged sulfonate group of AMPS in the chemical structures of X1, which interacted with the positively charged magnetite nanoparticles to increase the attractive forces that increase the magnetite agglomerations. Yet the wide distribution of the iron oxide nanoparticles can be clearly observed in the SEM images; the sizes of each single particle was calculated and was found to be ~24 nm. This observation agrees with Korotych’s group [38]. They demonstrated that the in situ synthesis of Fe_3_O_4_ particles in nanoreactors of thermosensitive copolymeric hydrogels (i.e., made of N-isopropylacrylamide (NIPAAm), acrylamide (AAm), and N,N′-methylenebisacrylamide (MBA) as crosslinkers permitted their stabilization, prevented nanoparticle aggregation, and allowed obtaining magnetic particles with an average size of about 20 nm (0.2% of MBA).

### 3.2. Swelling Curves

**Figure 4:**
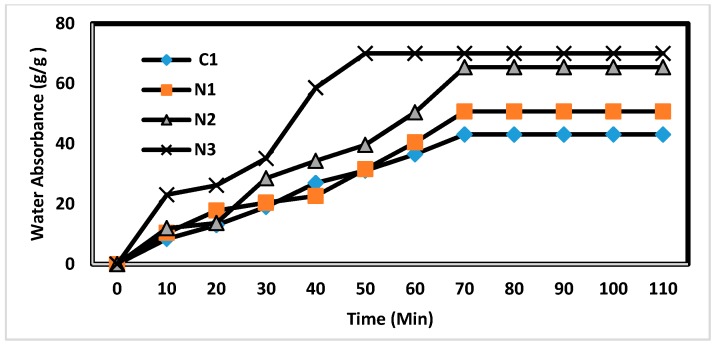
Shows the degree of swelling of the magnetic nanocomposite hydrogel samples (N1, N2, and N3) after 110 min.

**Figure 5:**
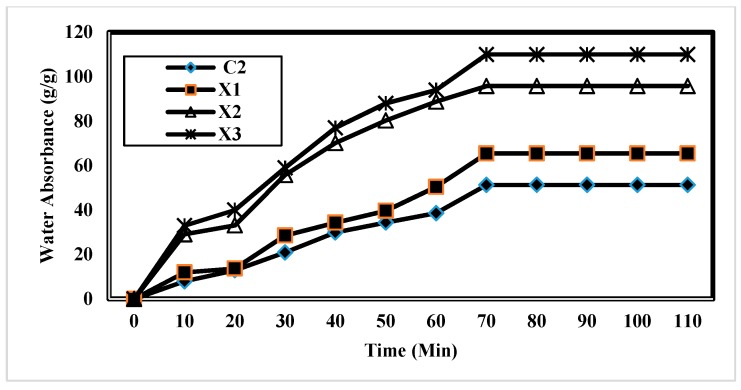
Shows the degree of swelling of the magnetic nanocomposite hydrogel samples (X1, X2, and X3 magnetite/terpolymer hybrids) after 110 min.

The swelling degree of the hydrogels and the nanocomposite hydrogel samples (C1, N1, N2, and N3) and magnetite/terpolymer hybrids (C2, X1, X2, and X3) was tested after being left for 110 min in water at room temperature. The results in the curves (Figure 4 and Figure 5) reveal that the water absorption has started with increasing the time, and then it reached constancy. Moreover, it showed that the absorption increased due to the increasing concentration of the acrylonitrile compared to the AMPs and acrylic acid ratio in Figure 4, and by increasing the concentrations of the acrylonitrile and acrylamide monomers in Figure 5. The equilibrium was achieved after 110 min (Tmax). The increasing AN concentrations (N2 and N3; Figure 4) increased its copolymerization with AMPS more than its copolymers with AA due the reactivity ratios data of the AMPS/AN, AA/AN, and AMPS/AA copolymers [39,40]. The reactivity ratios data ((r_AMPS_:r_AN_; 0.7:1.2), (r_AMPS_:r_AA_; 0.19:0.86), and (r_AA_:r_AN_; 3.07:0.27)) elucidate that the increasing of AN contents increases the incorporation of AMPS in the terpolymer hydrogels, which increases the swelling capacity. Moreover, the increasing of AN and AAm contents (X2 and X3; Figure 5) increase the amounts of AA and the amide ratios due to increasing reactivity of AA toward AAm (r_AAm_:r_AA_; 0.47:1.3) and AN (r_AA_:r_AN_; 3.07:0.27) [39,40]. This may be accredited to the reactivity ratio of acrylamide and acrylonitrile, which is more reactive in Figure 5 than AMPS and acrylic acid respectively, as shown in in Figure 4. Moreover, the nitrile and amide amounts increase and form a deeper network of the polymer and reduce the average molecular weight between the crosslinks, which causes—for example—a restricted relaxation of the polymeric chain. Due to the formation of denser networks, small cavities were produced that allowed the polymer chains to be stable by enhancing its collapse. In addition, these small cavities provide larger absorption surfaces, which allow the polymeric network to have a higher swelling rate. As a consequence of the greater absorption surface, the diffusion of the solvent molecules increases and penetrates through the network. The results also show that the water absorption of the magnetic nanocomposite was higher than that of the pure hydrogels in all the samples, and that was because the nanocomposite hydrogels forms a stiffer crosslinked network and produces smaller cavities, which enhances the collapse of the polymeric chain and causes it to be stable in microsize. In addition, it also provides larger absorption surfaces, which allows the polymeric nanonetwork to have a higher swelling rate. This observation shows significance with research done by Farag and Mohamed [41], where the results showed that the swelling capacity increased in the poly (acrylonitrile)-based hydrogels; moreover, by increasing its concentration, that of the acrylic acid-based hydrogels as well as the swelling ability decreased in the presence of high concentrations of the crosslinker. The incorporation of AMPS in the chemical structures of C1 (Figure 4) reduces the water absorbance of a terpolymer from 51.3 (C2 Figure 5) to 43.3 g/g (Figure 5). This was attributed to the presence of high contents of COOH groups of AA in the chemical structure of the C2 terpolymer more than the sulfonate group of the AMPS terpolymer (C1), which increases the water absorption capacities of C2, X1, X2, and X3 more than C1, N1, N2, and N3 magnetite/terpolymer hybrids.

### 3.3. Biological Activity Results

**Figure 6:**
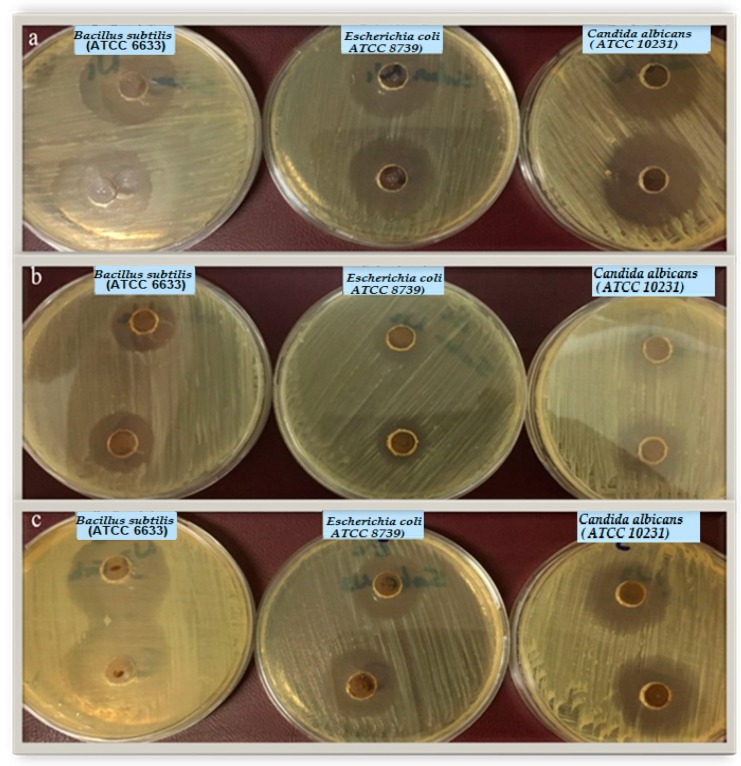
The agar well diffusion method of the magnetic nanocomposite hydrogel samples where (**a**) represents the agar well diffusion method using the X1 magnetite/terpolymer hybrid sample to test antimicrobial effect on bacterial and fungal strains (*Bacillus subtilis, E. coli,* and *Candida albicans*); (**b**) represents the agar well diffusion method using the X2 sample to test the antimicrobial effect; and (**c**) represents the agar well diffusion method using the X3 sample to test the antimicrobial effect.

The biological activity of iron oxide nanocomposite hydrogels (X1, X2, and X3 magnetite/terpolymer hybrids) against two Gram-positive bacteria, two Gram-negative bacterial strains, and one Candia strain were reported. All of the synthesized compounds have displayed broad spectrum antibacterial and anti-*Candida* activity with the clearing inhibition zones ranging from 19.3 to 36 mm in comparison to standard antimicrobial agents (see Figure 6 and Table 2). Moreover, all the iron oxide nanocomposite hydrogels (X1, X2, X3 magnetite/terpolymer hybrids) have demonstrated a higher antibacterial activity for the Gram-positive (23.5–36.0 nm) than for the Gram-negative bacteria (19.3–29 mm). This result may be attributed to the changing in the cell wall structure of Gram-positive bacteria in comparison to Gram-negative bacteria, as previously reported [41,42]. The X2 magnetite/terpolymer hybrid has shown the lowest biological activity in comparison to the X3 and X1 magnetite/terpolymer hybrids. The X3 magnetite/terpolymer hybrid has displayed the highest anti-Gram-positive bacterial activity followed by the X1 magnetite/terpolymer hybrid and X2 magnetite/terpolymer hybrid. Meanwhile, the biological activity of the iron oxide nanocomposite hydrogels (X1, X2, and X3 magnetite/terpolymer hybrid) were different against Gram-negative bacteria: X1 followed by the X3 and X2 magnetite/terpolymer hybrids.

These results were related to the network structure of the tested nanocomposite samples. They are credited mainly to the higher swelling ability of the nanocomposites, which was expected to improve the diffusion of the active ingredients inside the pathogens rather than allowing the pathogen to absorb the insoluble compounds on their surface, thus causing a disturbance to the enzyme activities that are responsible for the pathogen’s growth criteria [43]. Furthermore, iron oxide nanocomposite hydrogels (X1, X2, and X3 magnetite/terpolymer hybrids) have displayed anti-*Candida* activity with the highest activity for the X1 magnetite/terpolymer hybrid followed by the X3 and X2 magnetite/terpolymer hybrids.

The MICs, MBCs, and MFCs of the iron oxide nanocomposite hydrogels (X1, X2, and X3 magnetite/terpolymer hybrids) were listed (Table 3), where the X1 magnetite/terpolymer hybrid has exhibited the lowest MIC and MBC/MFC (15.6/62.5 ppm, 31.5/125 ppm, and 15.6/31.5) in comparison to the X3 magnetite/terpolymer hybrid (62.5/125 ppm, 62.5/250 ppm, and 31.2/62.5 ppm) and the X2 magnetite/terpolymer hybrid (125/250 ppm, 125/500 ppm and 62.5/125 ppm) against the Gram-positive bacteria, Gram-negative bacteria, and *Candida*, respectively. The explanation of these results may due to the presence of increased concentrations of acrylic acid moiety with reduced pH values in the drop after time. It’s known that low pH values stresses the cell by disrupting the cytoplasmic pH homeostasis, besides impeding the enzyme and transport system functions [44,45]. In addition, the long exposure of the microorganism to acids results in protein denaturation, DNA depurination, and damage in the membrane [46,47]. Small leaching acids such as the fatty acids are expected to attack the cell membrane [48,49,50], and it’s also known that they kill the bacteria by depending on the acid pH value and concentration. The high potential of the acidic material is shown due to some acids being antiviral and antifungal [51]. An example of an acidic material that doesn’t contain leaching acids is the ferulic acid copolymers activity against the *Aspergillus niger* [52].

The hypothesized antibacterial mechanism of the nanocomposite hydrogels may be attributed to their ability to penetrate into the cells of the microorganisms, preventing the cells’ growth by preventing the transformation of DNA to RNA to obtain a higher antibacterial activity. Meanwhile, the different inhibitory effect of the hydrogel nanocomposites may be due to the extent of the swell ability of the nanogels. The swelling property seems to improve the contact surface between the gel and the bacteria. Furthermore, it seems that the lower reactivity of acrylic acid compared to acrylonitrile or acrylamide, the higher the degree of freedom of the crosslinked acrylic acid chains that can fulfill their antibacterial activity while still covalently being immobilized into the network.

## 4. Conclusions

In conclusion, the synthesis of a novel superabsorbent terpolymer nanocomposite hydrogel through using the free radical copolymerization method based on the usage of AMPS, acrylamide, acrylonitrile, and acrylic acid monomers and iron oxide (Fe_3_O_4_) magnetic nanoparticles while using benzoyl peroxide as an initiator and ethylene glycol dimethacrylate (EGDMA) as a crosslinker was successful, as (C1, N1, N2, and N3) magnetite/terpolymer hybrids and (C2, X1, X2, and X3) magnetite/terpolymer hybrids were synthesized. The terpolymer nanocomposite hydrogels (X1, X2, X3 magnetite/terpolymer hybrids) showed better swelling results than the (N1, N2, N3 magnetite/terpolymer hybrids) nanocomposite hydrogels, and that’s due to the reactivity ratio of acrylamide and acrylonitrile, which is more reactive than the AMPS and acrylic acid, respectively, as well as due to the absence of the crosslinking agent in the samples. Furthermore, all the terpolymer nanocomposite hydrogels (X1, X2, and X3 magnetite/terpolymer hybrids) showed an antimicrobial activity, which may be due to their ability to penetrate into the cells of the microorganisms, preventing the cells’ growth by preventing the transformation of DNA to RNA to obtain a higher antibacterial activity. While within these hydrogel compounds, the X1 magnetite/terpolymer hybrids showed the highest activity due to the presence of increased concentrations of acrylic acid moiety with reduced pH values in the drop after time.

## Figures and Tables

**Table 1:** Illustration of the components of the prepared samples. AMPS: 2-acrylamido-2-methylpropane sulfonic acid, AAm: acrylamide, AN: acrylonitrile, AA: acrylic acid, EDGMA: ethylene glycol dimethacrylate, BP: benzoyl peroxide, Fe_3_O_4_: magnetic nanoparticles.

Sample Name	Monomers Recipe							
	AMPS (g)	AA (mL)	AN (mL)	AAm (g)	EDGMA (mL)	BP (g)	Fe_3_O_4_ (g)	Water mL
N1	0.52	0.72	0.53	-	0.177	0.15	0.195	50
N2	0.52	0.72	1.06	-	0.23	0.15	0.258	50
N3	0.52	0.72	2.12	-	0.33	0.15	0.369	50
C1	0.52	0.72	0.53	-	0.177	0.15	-	50
X1	-	0.72	0.53	0.71	0.196	0.15	0.216	50
X2	-	0.72	1.06	1.42	0.320	0.15	0.352	50
X3	-	0.72	2.12	2.84	0.568	0.15	0.625	50
C2	-	0.72	0.53	0.71	0.196	0.15	-	50

**Table 2:** The antimicrobial (antibacterial and antifungal) activity of the nanocomposite hydrogel samples (X1, X2, and X3 magnetite/terpolymer hybrids). The result was described as the mean of the inhibition zones diameter (mm) with standard deviations (SD).

Samples	*Bacillus subtilis*	*Escherichia coli*	*Candida albicans*
Inhibition Zone (mm)			
X1	32.6 ± 0.4	29.0 ± 1.0	29.0 ± 1.0
X2	23.5 ± 0.5	19.3 ± 0.9	21.5 ± 1.5
X3	36.0 ± 0.0	25.3 ± 0.4	26.5 ± 0.5
* AMC (100 ppm)	18		
* TE (100 ppm)		22	
* Flu (100 ppm)			17

* AMC, amoxicillin, TE, tetracycline, Flu, fluconazole.

**Table 3:** The minimum inhibitory concentrations (MICs), the minimum bactericidal/fungicidal concentrations (MBCs/MFCs) of the nanocomposite hydrogel samples (X1, X2, and X3 magnetite/terpolymer hybrids) against different standard bacterial and fungal strains. The result was represented as the mean of the samples’ concentrations (PPM).

Samples	*Bacillus subtilis*		*Escherichia coli*		*Candida albicans*	
	MIC (PPM)	MBC (PPM)	MIC (PPM)	MBC (PPM)	MIC (PPM)	MFC (PPM)
X1 MAGNETITE/TERPOLYMER HYBRIDE MAGNETITE/TERPOLYMER HYBRID	15.62	62.5	31.2	125	15.6	31.2
X2	125	250	125	500	62.5	125
X3	62.5	125	62.5	250	31.2	62.5

## References

[1] Wei L., Hu N., Zhang Y. (2010). Synthesis of polymer—Mesoporous silica nanocomposites. Materials.

[2] Datta K., Achari A., Eswaramoorthy M. (2013). Aminoclay: A functional layered material with multifaceted applications. J. Mater. Chem. A.

[3] Janas D., Boncel S., Koziol K.K. (2014). Electrothermal halogenation of carbon nanotube films. Carbon.

[4] Gaharwar A.K., Peppas N.A., Khademhosseini A. (2014). Nanocomposite hydrogels for biomedical applications. Biotechnol. Bioeng..

[5] Sharma G., Thakur B., Naushad M., Kumar A., Stadler F.J., Alfadul S.M., Mola G.T. (2018). Applications of nanocomposite hydrogels for biomedical engineering and environmental protection. Environ. Chem. Lett..

[6] Song F., Li X., Wang Q., Liao L., Zhang C. (2015). Nanocomposite hydrogels and their applications in drug delivery and tissue engineering. J. Biomed. Nanotechnol..

[7] Shin M.K., Spinks G.M., Shin S.R., Kim S.I., Kim S.J. (2009). Nanocomposite hydrogel with high toughness for bioactuators. Adv. Mater..

[8] Adhikari B., Biswas A., Banerjee A. (2012). Graphene oxide-based hydrogels to make metal nanoparticle-containing reduced graphene oxide-based functional hybrid hydrogels. ACS Appl. Mater. Interfaces.

[9] Goenka S., Sant V., Sant S. (2014). Graphene-based nanomaterials for drug delivery and tissue engineering. J. Control. Release.

[10] Tiwari A., Grailer J.J., Pilla S., Steeber D.A., Gong S. (2009). Biodegradable hydrogels based on novel photopolymerizable guar gum–methacrylate macromonomers for in situ fabrication of tissue engineering scaffolds. Acta Biomater..

[11] Madhumathi K., Kumar P.S., Abhilash S., Sreeja V., Tamura H., Manzoor K., Nair S., Jayakumar R. (2010). Development of novel chitin/nanosilver composite scaffolds for wound dressing applications. J. Mater. Sci. Mater. Med..

[12] Eid M., El-Arnaouty M., Salah M., Soliman E.-S., Hegazy E.-S.A. (2012). Radiation synthesis and characterization of poly (vinyl alcohol)/poly (N-vinyl-2-pyrrolidone) based hydrogels containing silver nanoparticles. J. Polym. Res..

[13] Varaprasad K., Raghavendra G.M., Jayaramudu T., Yallapu M.M., Sadiku R. (2017). A mini review on hydrogels classification and recent developments in miscellaneous applications. Mater. Sci. Eng. C.

[14] Mohan Y.M., Lee K., Premkumar T., Geckeler K.E. (2007). Hydrogel networks as nanoreactors: A novel approach to silver nanoparticles for antibacterial applications. Polymer.

[15] Tomar A., Garg G. (2013). Short review on application of gold nanoparticles. Glob. J. Pharmacol..

[16] Campbell J.L., Arora J., Cowell S.F. (2011). Quasi-cubic magnetite/silica core-shell nanoparticles as enhanced Mri contrast agents for cancer imaging. PLoS ONE.

[17] Eyvazzadeh N., Shakeri-Zadeh A., Fekrazad R. (2017). Gold-coated magnetic nanoparticle as a nanotheranostic agent for magnetic resonance imaging and photothermal therapy of cancer. Lasers Med. Sci..

[18] Pang Y., Wang C., Wang J. (2016). Fe3o4@Ag magnetic nanoparticles for microRNA capture and duplex-specific nuclease signal amplification based SERS detection in cancer cells. Biosens. Bioelectron..

[19] Heidaria F., Bahrololooma M.E., Vashaeeb D., Tayebicd T. (2015). In situ preparation of iron oxide nanoparticles in naturalhydroxyapatite/chitosan matrix for bone tissue engineering application. Ceram. Int..

[20] Iconaru S.L., Prodan A.M., Coustumer P.L., Predoi D. (2013). Synthesis and antibacterial and antibiofilm activity of iron oxide glycerol nanoparticles obtained by coprecipitation method. J. Chem..

[21] Behera S.S., Patra J.K., Pramanik K., Panda N., Thatoi H. (2012). Characterization and evaluation of antibacterial activities of chemically synthesized iron oxide nanoparticles. World J. Nano Sci. Eng..

[22] Harifi T., Montazer M. (2014). In situ synthesis of iron oxide nanoparticles on polyester fabric utilizing color, magnetic, antibacterial and sono-Fenton catalytic properties. J. Mater. Chem. B.

[23] Konwar A., Kalita S., Kotoky J., Chowdhury D. (2016). Chitosan–iron oxide coated graphene oxide nanocomposite hydrogel: A robust and soft antimicrobial biofilm. ACS Appl. Mater. Interfaces.

[24] Akl Z.F., El-Saeed S.M., Atta A.M. (2016). In-situ synthesis of magnetite acrylamide amino-amidoxime nanocomposite adsorbent for highly efficient sorption of U (VI) ions. J. Ind. Eng. Chem..

[25] Atta A.M., Gafer A.K., Al-Lohedan H.A., Abdullah M.M.S., Ezzat A.O. (2019). Preparation of magnetite and silver poly(2-acrylamido-2-methyl propane sulfonic acid-*co*-acrylamide) nanocomposites for adsorption and catalytic degradation of methylene blue water pollutant. Polym. Int..

[26] Al-Hussain S.A., Ezzat A.O., Gaffer A.K., Atta A.M. (2018). Removal of organic water pollutant using magnetite nanomaterials embedded with ionic copolymers of 2-acrylamido-2-methylpropane sodium sulfonate cryogels. Polym. Int..

[27] Bal A., Çepni F., Çakir Ö., Acar I., Güçlü G. (2015). Synthesis and characterization of copolymeric and terpolymeric hydrogel-silver nanocomposites based on acrylic acid, acrylamide and itaconic acid: Investigation of their antibacterial activity against gram-negative bacteria. Braz. J. Chem. Eng..

[28] Qavi S., Pourmahdian S., Eslami H. (2014). Acrylamide hydrogels preparation via free radical crosslinking copolymerization: Kinetic study and morphological investigation. J. Macromol. Sci. Part A.

[29] Balouiri M., Sadiki M., Ibnsouda S.K. (2016). Methods for in vitro evaluating antimicrobial activity: A review. J. Pharm. Anal..

[30] Labena A., Kabel K., Farag R. (2016). One-pot synthesize of dendritic hyperbranched PAMAM and assessment as a broad spectrum antimicrobial agent and anti-biofilm. Mater. Sci. Eng. C.

[31] Wiegand I., Hilpert K., Hancock R.E. (2008). Agar and broth dilution methods to determine the minimal inhibitory concentration (MIC) of antimicrobial substances. Nat. Protoc..

[32] Modrow S., Falke D., Truyen U., Schätzl H. (2013). Molecular Virology.

[33] Amsterdam D. (1996). Susceptibility testing of antimicrobials in liquid media. Antibiot. Lab. Med..

[34] Clinical and Laboratory Standards Institute (2006). Methods for dilution antimicrobial susceptibility tests for bacteria that grow aerobically. Approv. Stand..

[35] Rukayadi Y., Han S., Yong D., Hwang J.-K. (2010). In vitro antibacterial activity of panduratin A against enterococci clinical isolates. Biol. Pharm. Bull..

[36] Kebbekus B.B., Harwood L.M., Claridge T.D.W. (1997). Introduction to Organic Spectroscopy.

[37] Kim H.C., Gao X., Jayaramudu T., Kang J., Kim J. (2017). Optical and electro-active properties of polyacrylamide/CNC composite hydrogels. Mater. Sci..

[38] Nakamura T., Tamura A., Murotani H., Oishi M., Jinji Y., Matsuishi K., Nagasaki Y. (2010). Large payloads of gold nanoparticles into the polyamine network core of stimuli-responsive PEGylated nanogels for selective and noninvasive cancer photothermal therapy. Nanoscale.

[39] Bajai P., Paliwal D.K., Gupta A.K. (1993). Acrylonitrile-acrylic acids copolymers. 1. synthesis and characterization. J. Appl. Polym. Sci..

[40] Chapiro A. (1989). Influence of solvents on apparent reactivity ratios in the free radical copolymerization of polar monomers. Eur. Polym. J..

[41] Farag R., Mohamed R. (2012). Synthesis and characterization of carboxymethyl chitosan nanogels for swelling studies and antimicrobial activity. Molecules.

[42] Kenawy E.-R., Worley S., Broughton R. (2007). The chemistry and applications of antimicrobial polymers: A state-of-the-art review. Biomacromolecules.

[43] Gratzl G., Paulik C., Hild S., Guggenbichler J.P., Lackner M. (2014). Antimicrobial activity of poly (acrylic acid) block copolymers. Mater. Sci. Eng. C.

[44] Krulwich T.A., Sachs G., Padan E. (2011). Molecular aspects of bacterial pH sensing and homeostasis. Nat. Rev. Microbiol..

[45] Zollfrank C., Gutbrod K., Wechsler P., Guggenbichler J.P. (2012). Antimicrobial activity of transition metal acid MoO3 prevents microbial growth on material surfaces. Mater. Sci. Eng. C.

[46] Foster J.W. (2004). *Escherichia coli* acid resistance: Tales of an amateur acidophile. Nat. Rev. Microbiol..

[47] Goddard J.M., Hotchkiss J. (2007). Polymer surface modification for the attachment of bioactive compounds. Prog. Polym. Sci..

[48] Matche R., Kulkarni G., Raj B. (2006). Modification of ethylene acrylic acid film for antimicrobial activity. J. Appl. Polym. Sci..

[49] Bassolé I.H.N., Juliani H.R. (2012). Essential oils in combination and their antimicrobial properties. Molecules.

[50] Lang G., Buchbauer G. (2012). A review on recent research results (2008–2010) on essential oils as antimicrobials and antifungals. A review. Flavour Fragr. J..

[51] Edris A.E. (2007). Pharmaceutical and therapeutic potentials of essential oils and their individual volatile constituents: A review. Phytother. Res..

[52] Iemma F., Puoci F., Curcio M., Parisi O.I., Cirillo G., Spizzirri U.G., Picci N. (2010). Ferulic acid as a comonomer in the synthesis of a novel polymeric chain with biological properties. J. Appl. Polym. Sci..

